# Supplementary material for: Modeling Structural Constraints on Protein Evolution via Side-Chain Conformational States
Source: Mol Biol Evol. 2019 May 22;36(9):2086–103. doi: 10.1093/molbev/msz122 (PMC6736381; doi:10.1093/molbev/msz122)
Supplement: msz122_Supplementary_Data [file msz122_supplementary_data.zip › RAM55_supplementary_figures.pdf]

## Supplementary Tables

| Taxa | Scaling factor | RAM55 | RUM20 | LG | LGexp | LGbyfreq-exp | LG+F |
|---|---|---|---|---|---|---|---|
| 4 | 0.1 | 1.00 | 0.00 | 0.0 | 0.0 | 0.0 | 0.0 |
| 4 | 0.2 | 1.00 | 0.00 | 0.0 | 0.0 | 0.0 | 0.0 |
| 4 | 0.5 | 1.00 | 0.00 | 0.0 | 0.0 | 0.0 | 0.0 |
| 4 | 0.7 | 1.00 | 0.00 | 0.0 | 0.0 | 0.0 | 0.0 |
| 4 | 1.2 | 1.00 | 0.00 | 0.0 | 0.0 | 0.0 | 0.0 |
| 4 | 1.5 | 0.96 | 0.04 | 0.0 | 0.0 | 0.0 | 0.0 |
| 4 | 1.7 | 0.99 | 0.01 | 0.0 | 0.0 | 0.0 | 0.0 |
| 4 | 2.0 | 0.96 | 0.04 | 0.0 | 0.0 | 0.0 | 0.0 |
| 4 | 2.5 | 0.93 | 0.07 | 0.0 | 0.0 | 0.0 | 0.0 |
| 4 | 3.0 | 0.77 | 0.23 | 0.0 | 0.0 | 0.0 | 0.0 |
| 8 | 0.1 | 1.00 | 0.00 | 0.0 | 0.0 | 0.0 | 0.0 |
| 8 | 0.2 | 1.00 | 0.00 | 0.0 | 0.0 | 0.0 | 0.0 |
| 8 | 0.5 | 1.00 | 0.00 | 0.0 | 0.0 | 0.0 | 0.0 |
| 8 | 0.7 | 1.00 | 0.00 | 0.0 | 0.0 | 0.0 | 0.0 |
| 8 | 1.2 | 0.99 | 0.01 | 0.0 | 0.0 | 0.0 | 0.0 |
| 8 | 1.5 | 0.99 | 0.01 | 0.0 | 0.0 | 0.0 | 0.0 |
| 8 | 1.7 | 0.99 | 0.01 | 0.0 | 0.0 | 0.0 | 0.0 |
| 8 | 2.0 | 0.96 | 0.04 | 0.0 | 0.0 | 0.0 | 0.0 |
| 8 | 2.5 | 0.86 | 0.14 | 0.0 | 0.0 | 0.0 | 0.0 |
| 8 | 3.0 | 0.89 | 0.11 | 0.0 | 0.0 | 0.0 | 0.0 |
| 16 | 0.1 | 1.00 | 0.00 | 0.0 | 0.0 | 0.0 | 0.0 |
| 16 | 0.2 | 1.00 | 0.00 | 0.0 | 0.0 | 0.0 | 0.0 |
| 16 | 0.5 | 1.00 | 0.00 | 0.0 | 0.0 | 0.0 | 0.0 |
| 16 | 0.7 | 1.00 | 0.00 | 0.0 | 0.0 | 0.0 | 0.0 |
| 16 | 1.2 | 1.00 | 0.00 | 0.0 | 0.0 | 0.0 | 0.0 |
| 16 | 1.5 | 1.00 | 0.00 | 0.0 | 0.0 | 0.0 | 0.0 |
| 16 | 1.7 | 0.95 | 0.05 | 0.0 | 0.0 | 0.0 | 0.0 |
| 16 | 2.0 | 0.95 | 0.05 | 0.0 | 0.0 | 0.0 | 0.0 |
| 16 | 2.5 | 0.84 | 0.16 | 0.0 | 0.0 | 0.0 | 0.0 |
| 16 | 3.0 | 0.80 | 0.20 | 0.0 | 0.0 | 0.0 | 0.0 |
| 32 | 0.1 | 1.00 | 0.00 | 0.0 | 0.0 | 0.0 | 0.0 |
| 32 | 0.2 | 1.00 | 0.00 | 0.0 | 0.0 | 0.0 | 0.0 |
| 32 | 0.5 | 1.00 | 0.00 | 0.0 | 0.0 | 0.0 | 0.0 |
| 32 | 0.7 | 1.00 | 0.00 | 0.0 | 0.0 | 0.0 | 0.0 |
| 32 | 1.2 | 1.00 | 0.00 | 0.0 | 0.0 | 0.0 | 0.0 |
| 32 | 1.5 | 1.00 | 0.00 | 0.0 | 0.0 | 0.0 | 0.0 |
| 32 | 1.7 | 1.00 | 0.00 | 0.0 | 0.0 | 0.0 | 0.0 |
| 32 | 2.0 | 1.00 | 0.00 | 0.0 | 0.0 | 0.0 | 0.0 |
| 32 | 2.5 | 1.00 | 0.00 | 0.0 | 0.0 | 0.0 | 0.0 |
| 32 | 3.0 | 0.98 | 0.02 | 0.0 | 0.0 | 0.0 | 0.0 |
| 64 | 0.1 | 1.00 | 0.00 | 0.0 | 0.0 | 0.0 | 0.0 |
| 64 | 0.2 | 1.00 | 0.00 | 0.0 | 0.0 | 0.0 | 0.0 |
| 64 | 0.5 | 1.00 | 0.00 | 0.0 | 0.0 | 0.0 | 0.0 |
| 64 | 0.7 | 1.00 | 0.00 | 0.0 | 0.0 | 0.0 | 0.0 |
| 64 | 1.2 | 1.00 | 0.00 | 0.0 | 0.0 | 0.0 | 0.0 |
| 64 | 1.5 | 1.00 | 0.00 | 0.0 | 0.0 | 0.0 | 0.0 |
| 64 | 1.7 | 1.00 | 0.00 | 0.0 | 0.0 | 0.0 | 0.0 |
| 64 | 2.0 | 1.00 | 0.00 | 0.0 | 0.0 | 0.0 | 0.0 |
| 64 | 2.5 | 1.00 | 0.00 | 0.0 | 0.0 | 0.0 | 0.0 |
| 64 | 3.0 | 1.00 | 0.00 | 0.0 | 0.0 | 0.0 | 0.0 |

**Supplementary Table 1:** Best model (AIC) for each category of simulated alignment. 1000-site alignments are simulated under the RAM55 model and various randomly-generated reference phylogenies (4,8,16,32 and 64 taxa) scaled according to a set of scaling factors. For each phylogeny and scaling factor pair the table reports the proportion of 100 replicates where each model achieves the lowest AIC (or state-corrected AIC, see *Log-likelihood comparison across models*) when compared against all other models.

| Taxa | RAM55 joint | LG joint | RAM55 marginal | LG marginal |
|---|---|---|---|---|
| Q71VM4/147-186 | 75.15% | 76.05% | 72.75% | 72.16% |
| P52292/410-447 | 76.35% | 75.75% | 73.05% | 73.05% |
| P52294/158-197 | 80.84% | 80.24% | 76.95% | 76.35% |
| P52293/251-279 | 81.14% | 80.84% | 78.44% | 77.55% |
| Q9C2K9/158-194 | 89.22% | 89.82% | 81.74% | 81.44% |
| O60684/113-154 | 88.92% | 88.32% | 85.03% | 85.03% |
| P35222/229-262 | 89.22% | 89.22% | 89.52% | 89.22% |
| P25054/649-689 | 88.92% | 88.32% | 89.22% | 89.52% |
| Q02821/372-411 | 97.01% | 97.01% | 97.31% | 96.71% |
| Q02248/229-262 | 96.41% | 96.41% | 97.01% | 96.41% |
| Q99959/386-423 | 95.51% | 95.51% | 95.81% | 95.81% |
| O60716/440-481 | 96.41% | 96.41% | 97.31% | 97.31% |
| O44326/361-402 | 99.40% | 99.10% | 99.40% | 99.40% |

**Supplementary Table 2:** Empirical amino acid reconstruction accuracy. Amino acid state reconstruction from the PF00514/$\beta$-catenin-like repeat alignment using RAM55 or LG and either the joint or marginal reconstruction algorithms. Scores represent the percentage of sites correctly reconstructed. Reconstruction was limited to terminal nodes using the LLO approach (see *Ancestral state reconstruction*), due to lack of a reference for internal sequences.

## Supplementary Figures

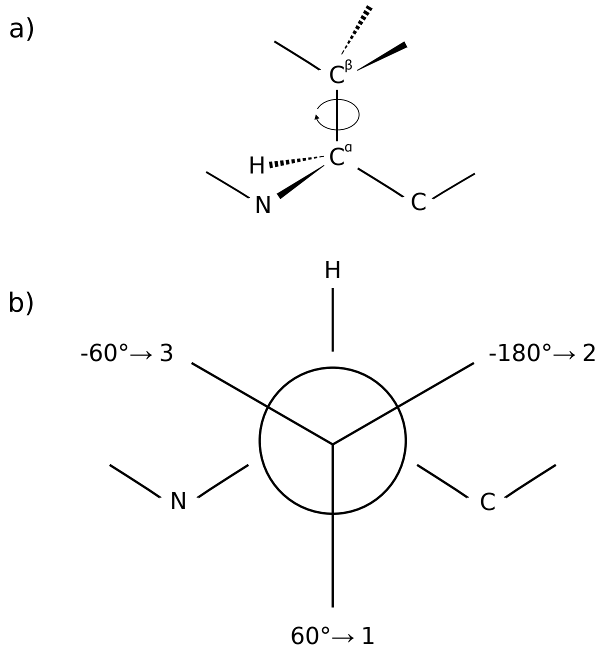


**Supplementary Figure 1:** Definition of rotamer configurations. **(a)** A $\chi_1$ rotamer configuration is defined by the dihedral angle generated by the rotation of the $C^\alpha$ - $C^\beta$ bond (curved arrow). **(b)** The three stable configurations correspond to specific $\chi_1$ dihedral angle values: $\sim 60^\circ$for configuration 1, $\sim -180^\circ$for configuration 2 and $\sim -60^\circ$for configuration 3. (See also Fig. 1 and Table 1)

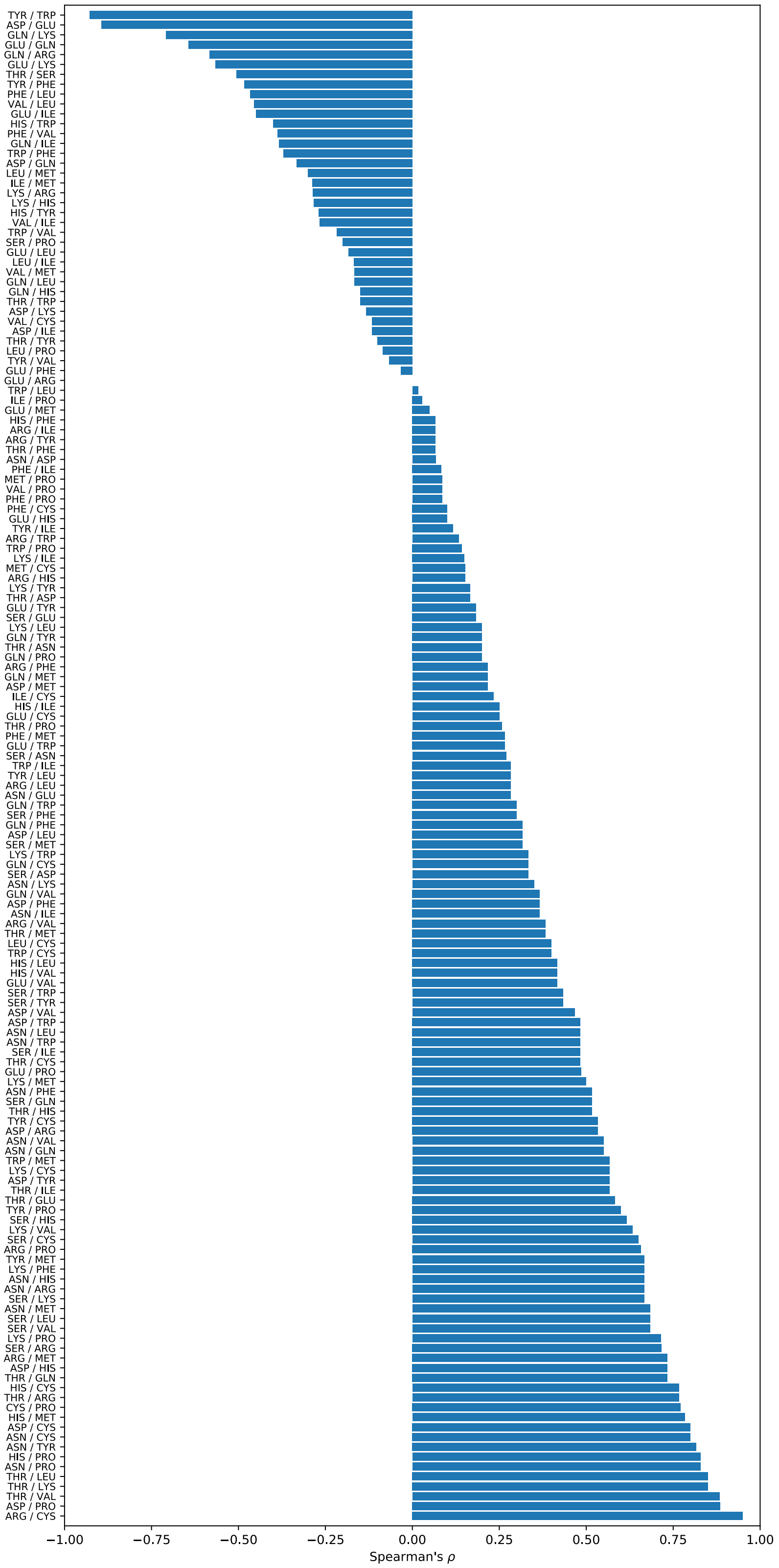


**Supplementary Figure 2:** Correlation between exchangeability and the overlap between $(\phi,\psi)$ distributions. For each amino acid pair (excluding alanine and glycine), the correlation between the exchangeabilities of their $\chi_1$ configurations and the overlap between their Ramachandran probability distributions is shown. The preponderance of positive correlations indicates that, for most amino acid pairs, there is a tendency to exchange between side-chain geometries that accommodate similar backbone geometries.

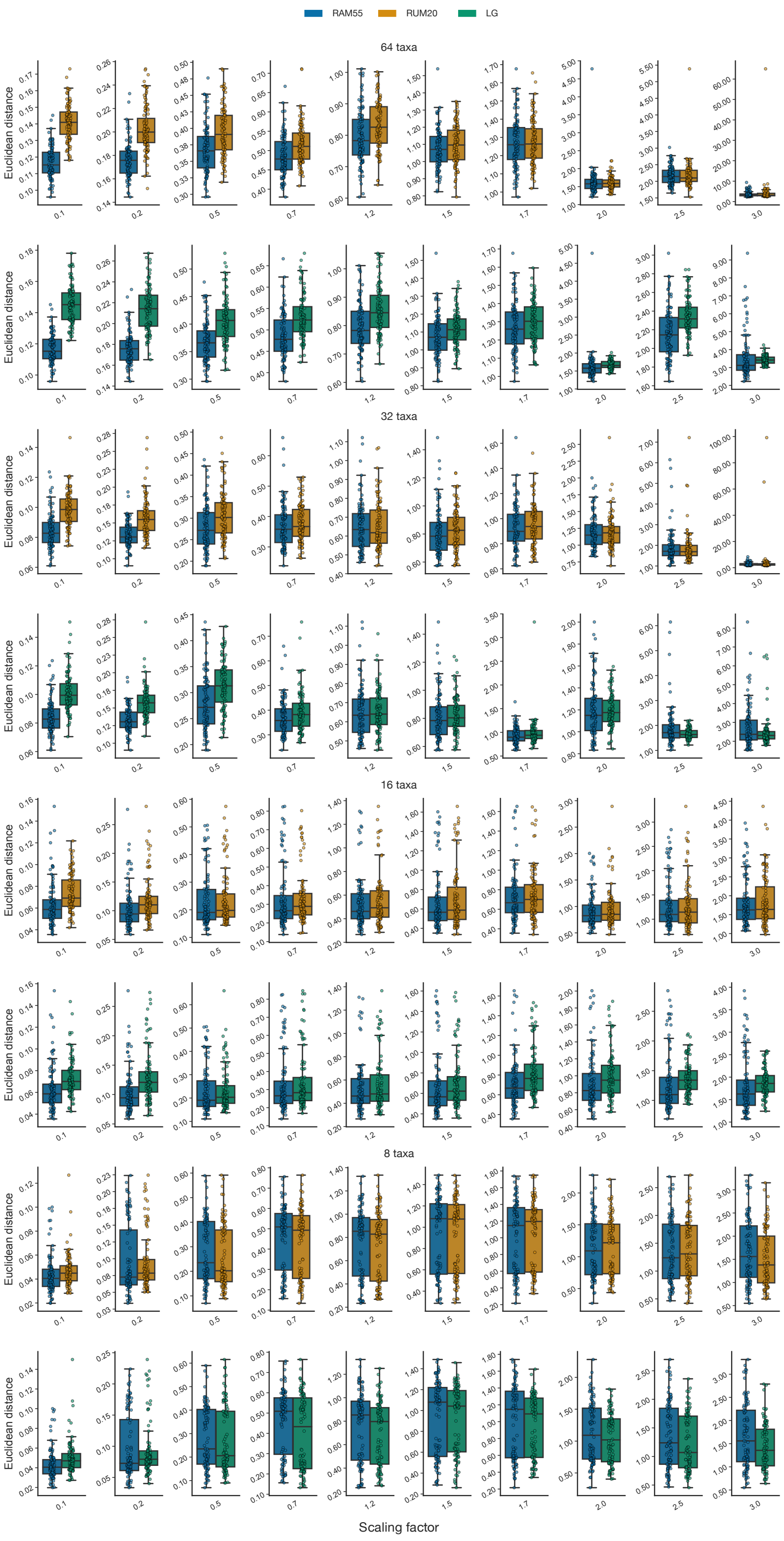


**Supplementary Figure 3:** Tree inference accuracy. Rotasequence alignments (200 sites, 100 replicates per scaling) are simulated under RAM55 and the trees in Sup. Fig. 11, scaled according to the factors on the $x$-axis. RUM20, LG and RAM55 itself are then used to perform inference over the simulated rotasequence alignments (or masked amino acid alignments for RAM20 and LG) and the resulting trees are compared to the original phylogeny in terms of Euclidean distance. RAM55 can infer trees that are closer to the original as shown by distance distributions and medians shifted towards lower values.

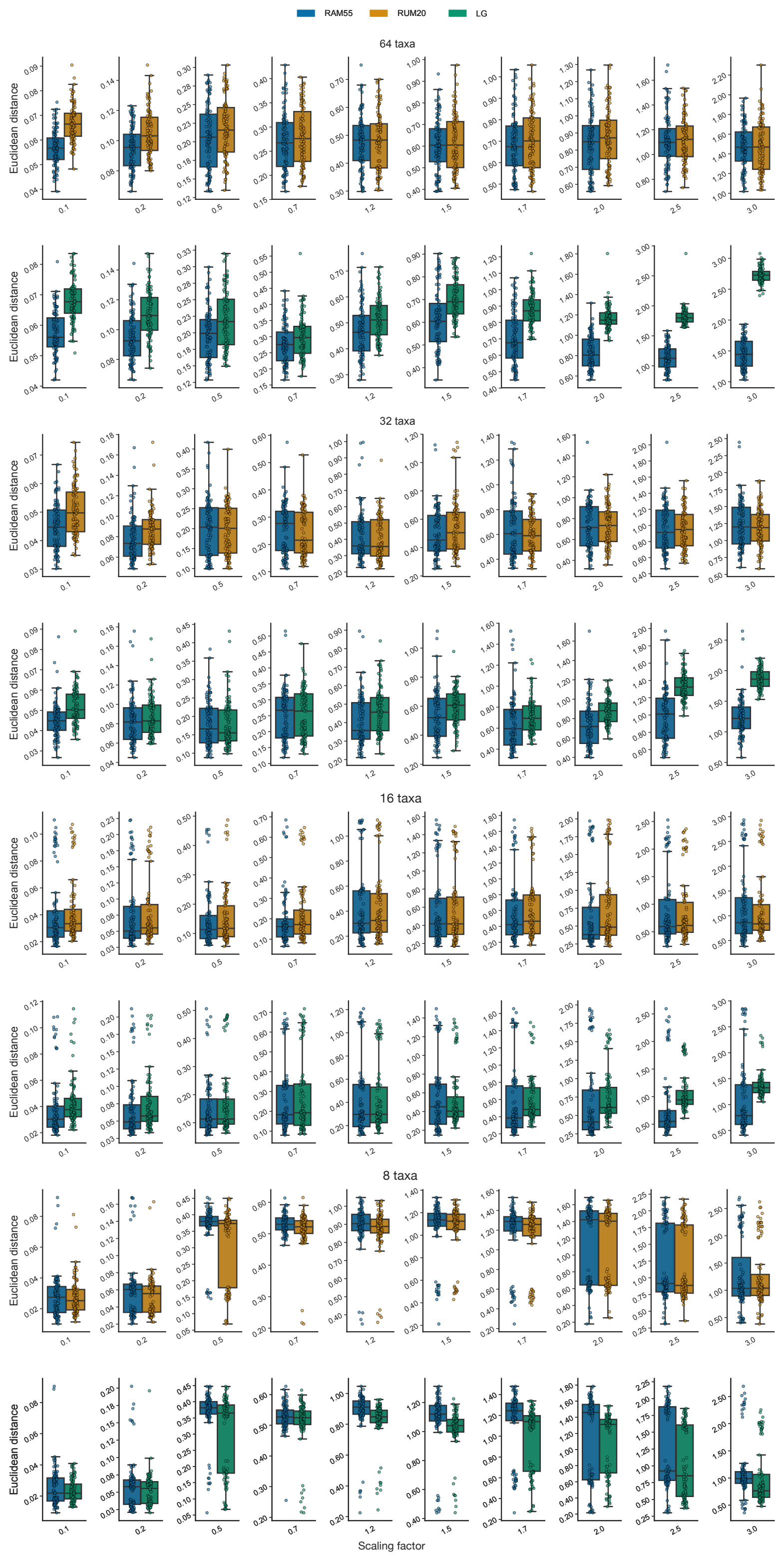


**Supplementary Figure 4:** Tree inference accuracy. As Sup. Fig. 3, except simulated alignments each contain 1000 sites.

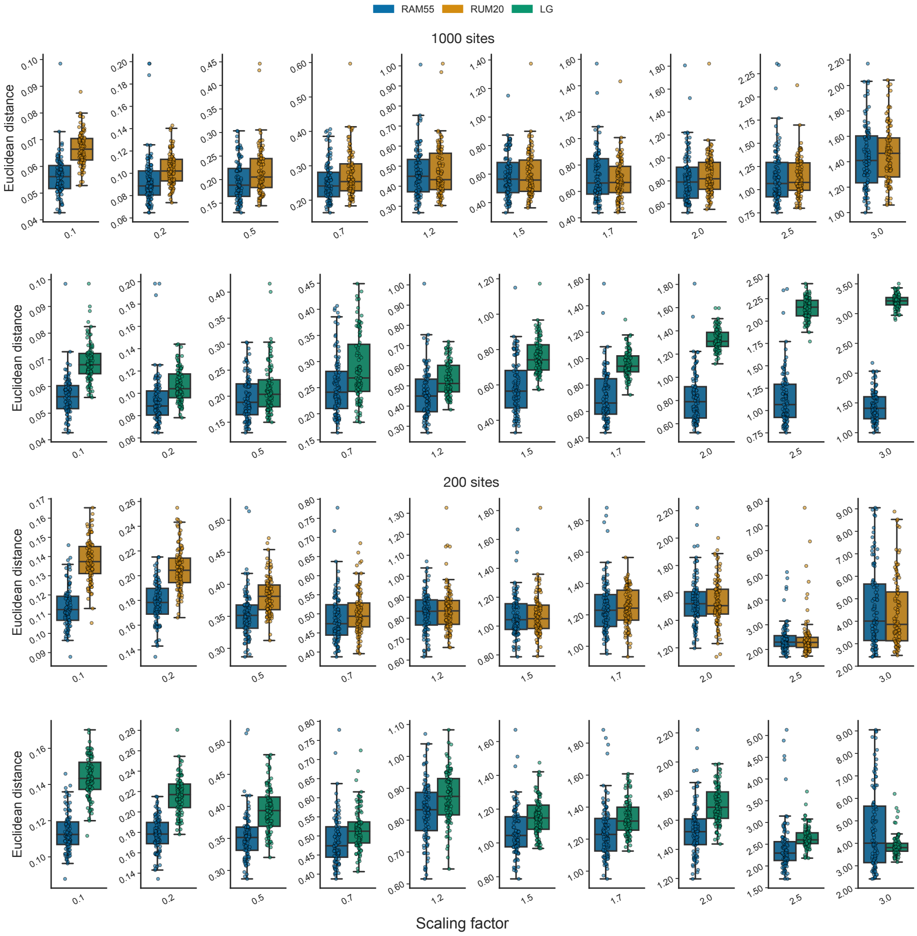


**Supplementary Figure 5:** Tree inference accuracy. As Sup. Fig. 3, except simulated alignments are generated under RAM55 and a pruned version of the Ensembl-compara tree (see *Tree generation and alignment simulation* and Sup. Files), scaled according to the factors on the $x$-axis.

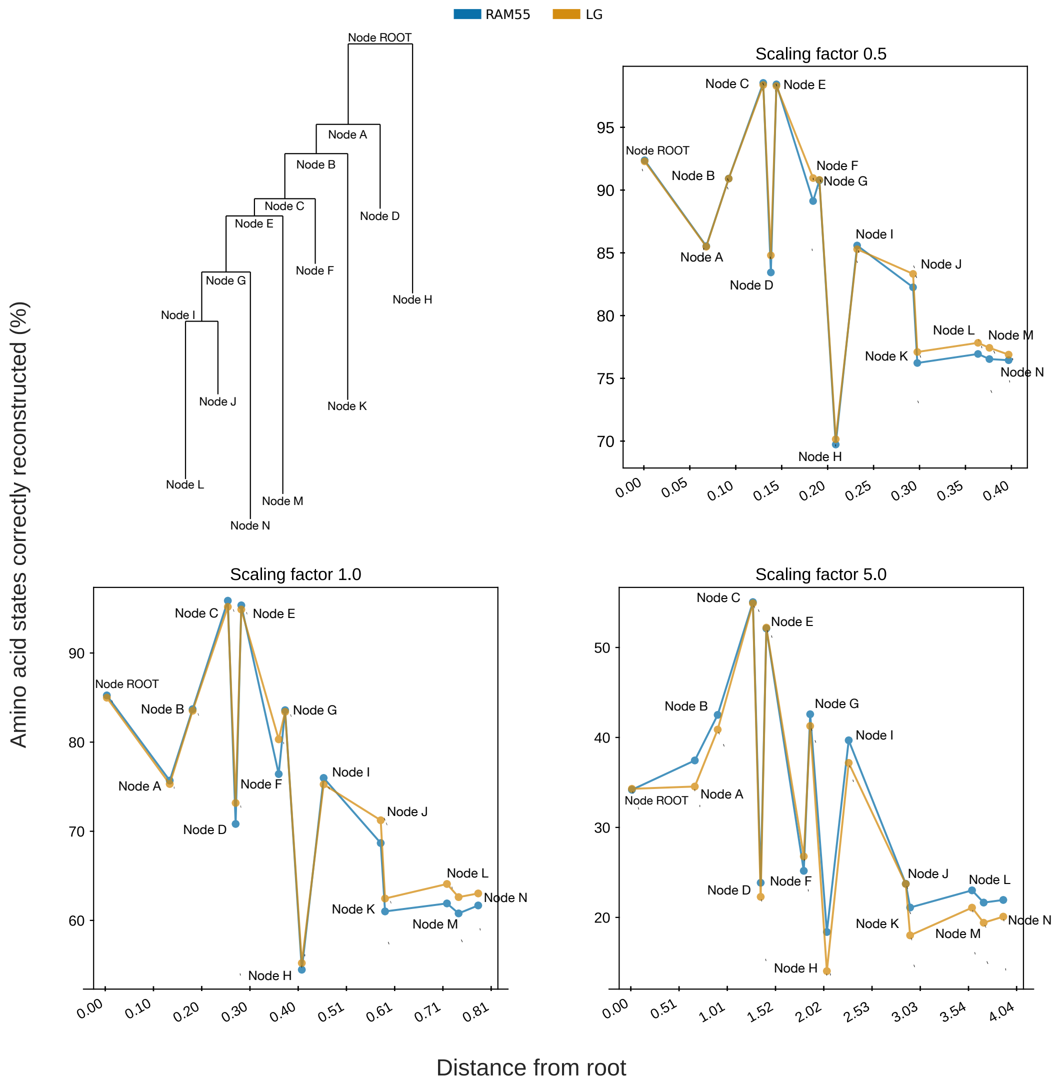


**Supplementary Figure 6:** Marginal reconstruction of inferred amino acid accuracy. Amino acid states inferred using marginal reconstruction from rotasequence alignments (200 sites, 8 taxa, 100 replicates per scaling) simulated under RAM55 using our 8-taxon reference phylogeny and scaling its branches according to the factor reported for each sub-plot. The marginal reconstruction algorithm is employed along with RAM55 and our 8-taxa phylogeny to reconstruct internal and terminal nodes' rotasequences which are then masked to obtain amino acid sequences (in blue, see *Ancestral state reconstruction*). The equivalent procedure is then repeated using LG on masked alignments (in orange). Each data point indicates (on the $y$-axis) the mean percentage of amino acid states correctly reconstructed from 100 replicates for a given reconstructed node, against its distance from the root node ($x$-axis).

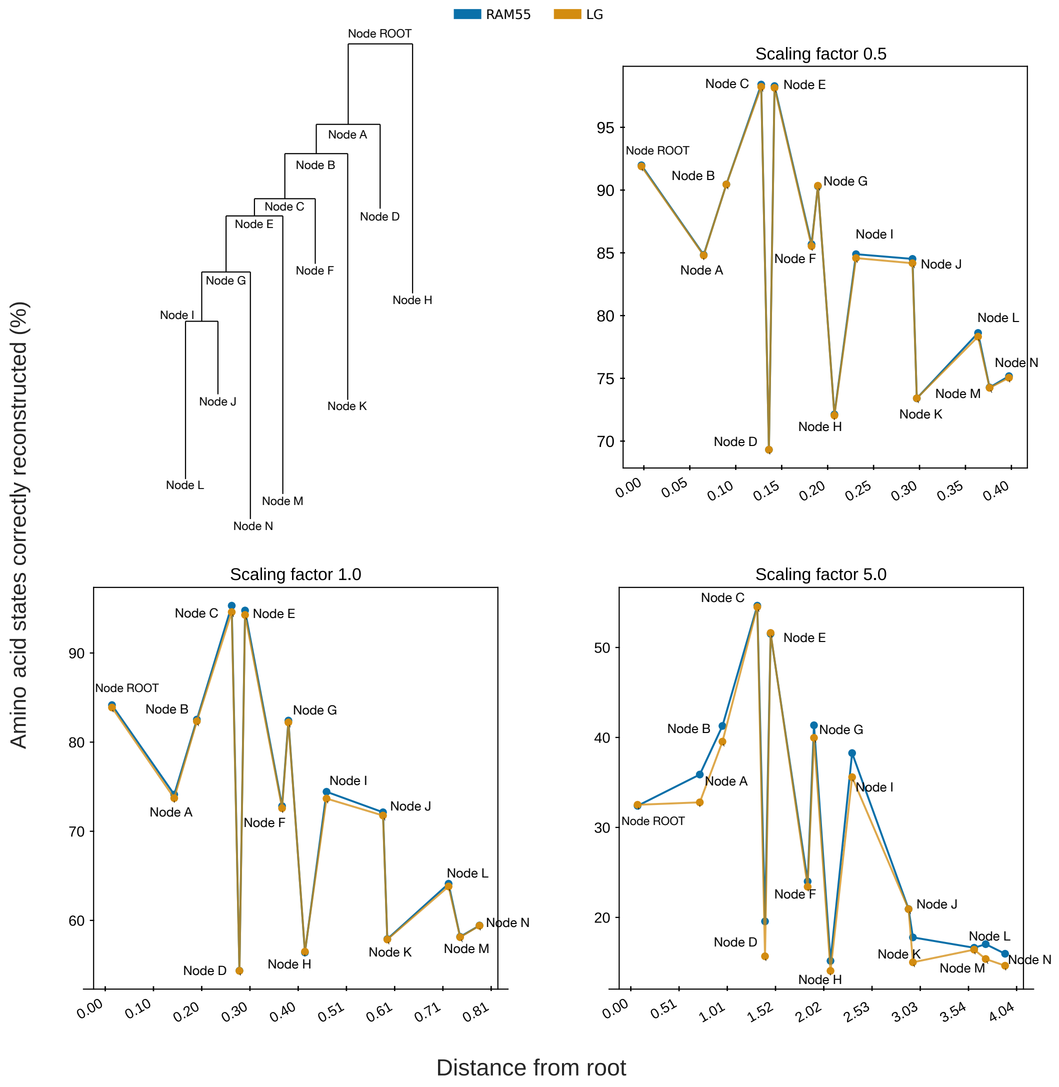


**Supplementary Figure 7:** Joint reconstruction of inferred amino acid accuracy. As Sup. Fig. 6, except joint reconstruction is used in place of marginal reconstruction. Marginal and joint reconstruction methods perform about equally in our studies.

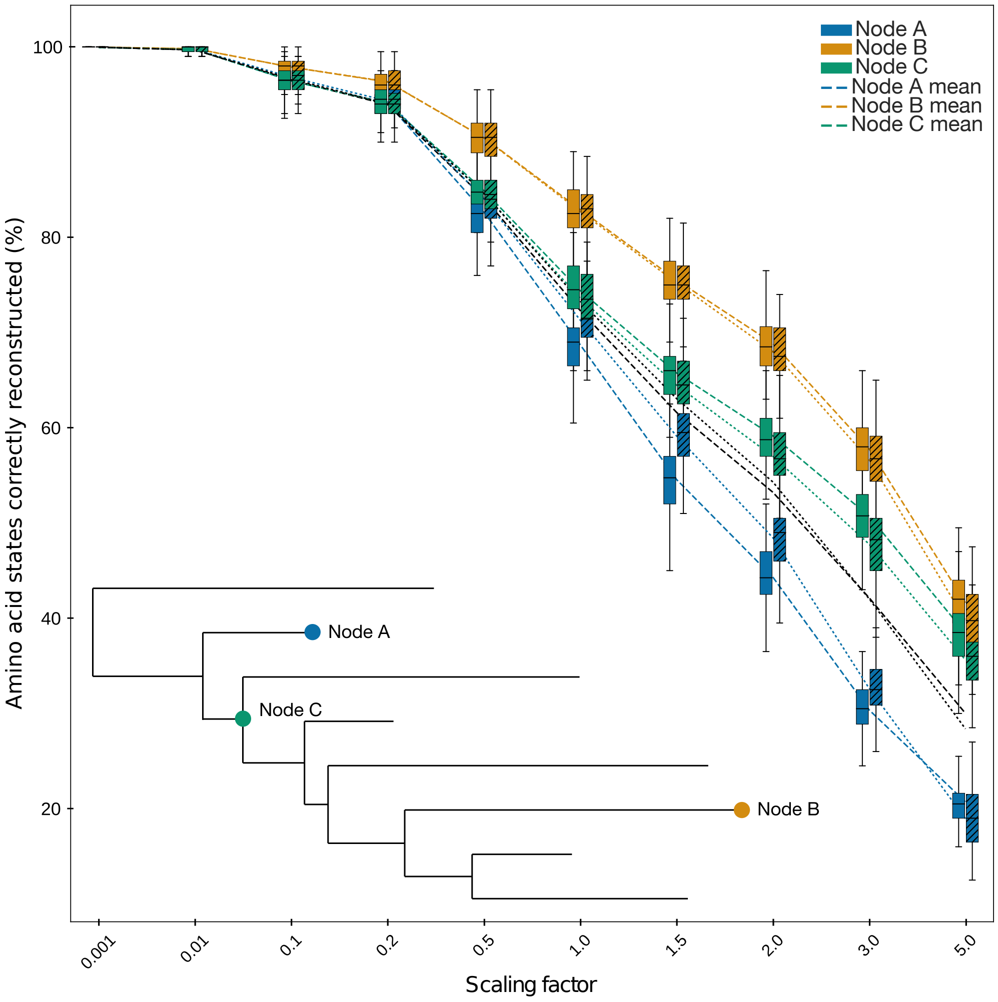


**Supplementary Figure 8:** Amino acid state reconstruction accuracy distributions. Ancestral amino acid states are inferred by marginal reconstruction from the same rotasequence alignments as in Fig. 8 using RAM55 and our 8-taxon reference phylogeny, at internal (C) and terminal (A, B) nodes. The same procedure is then repeated using LG on masked alignments (hatched). The $y$-axis report the percentage of sites correctly reconstructed for each inferred sequence. Each box-plot contains results from 100 simulation replicates for a given node. Marginal and joint reconstruction methods perform about equally in our studies.

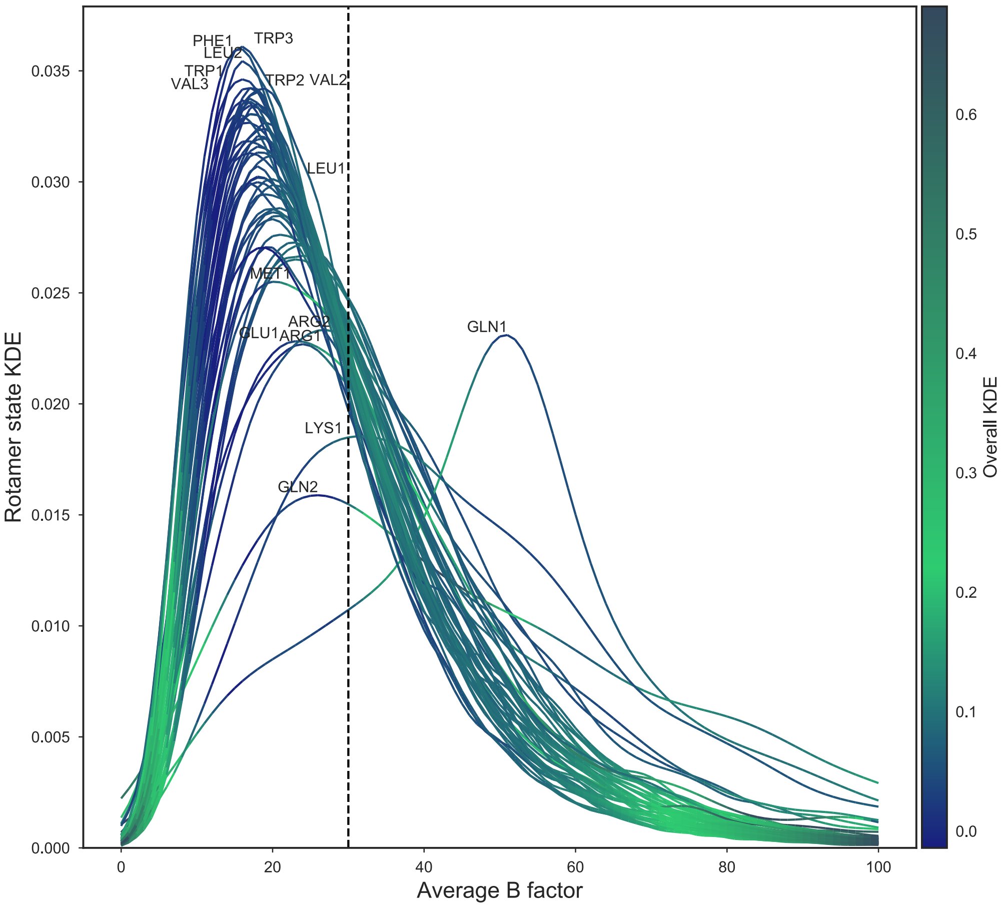


**Supplementary Figure 9:** Structural data quality distributions across residues. Kernel density estimate (KDE) for the average B-factor for each rotamer state (excluding alanine and glycine) across all residues in our unfiltered dataset. Average B-factor is computed over the four atoms ($N$, $C^{\alpha}$, $C^{\beta}$ and $C^{\gamma}$ for most residues) that constitute the dihedral angle defining the $\chi_1$ rotamer configuration. A threshold of B-factor $<30$ is then applied (dashed line) to ensure only highly reliable atomic coordinates are used to assign rotamer states. Only outlier density plots are labelled, for clarity; the color scheme represents overall plot density distribution at each point along the $x$-axis. Areas under the curves to the right of the dashed line indicates the proportions of each rotamer state in our alignments that are removed by the B-factor filtering.

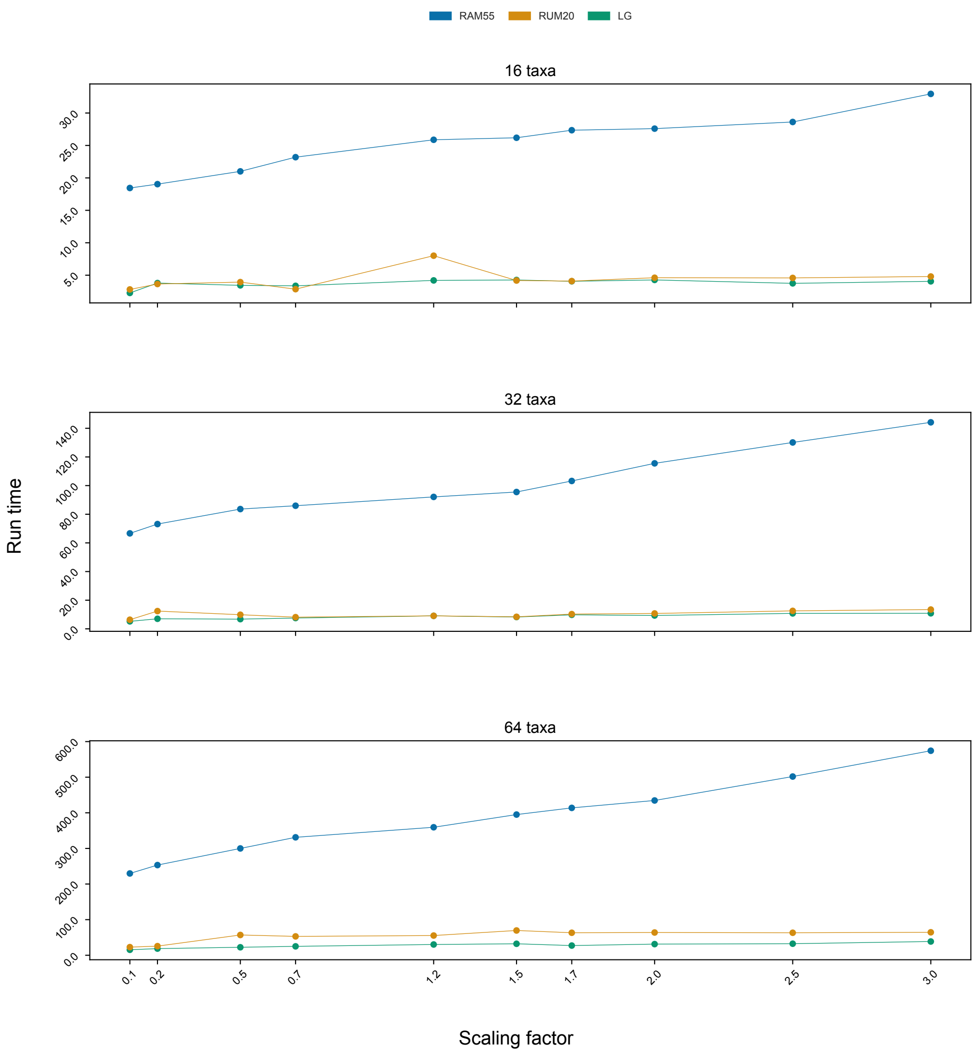


**Supplementary Figure 10:** Run time comparisons. Rotasequence alignments (200 sites, 100 replicates per scaling factor) were simulated using RAM55 and the 16-, 32- and 64-taxon trees of Sup. Fig. 11, scaled according to the factors shown on the $x$-axis. The plots report mean run times (in seconds) for ML inference analysis of these rotasequence alignment data under the RUM20, LG and RAM55 models in RAxML-NG (using masked alignments for the RUM20 and LG analyses).

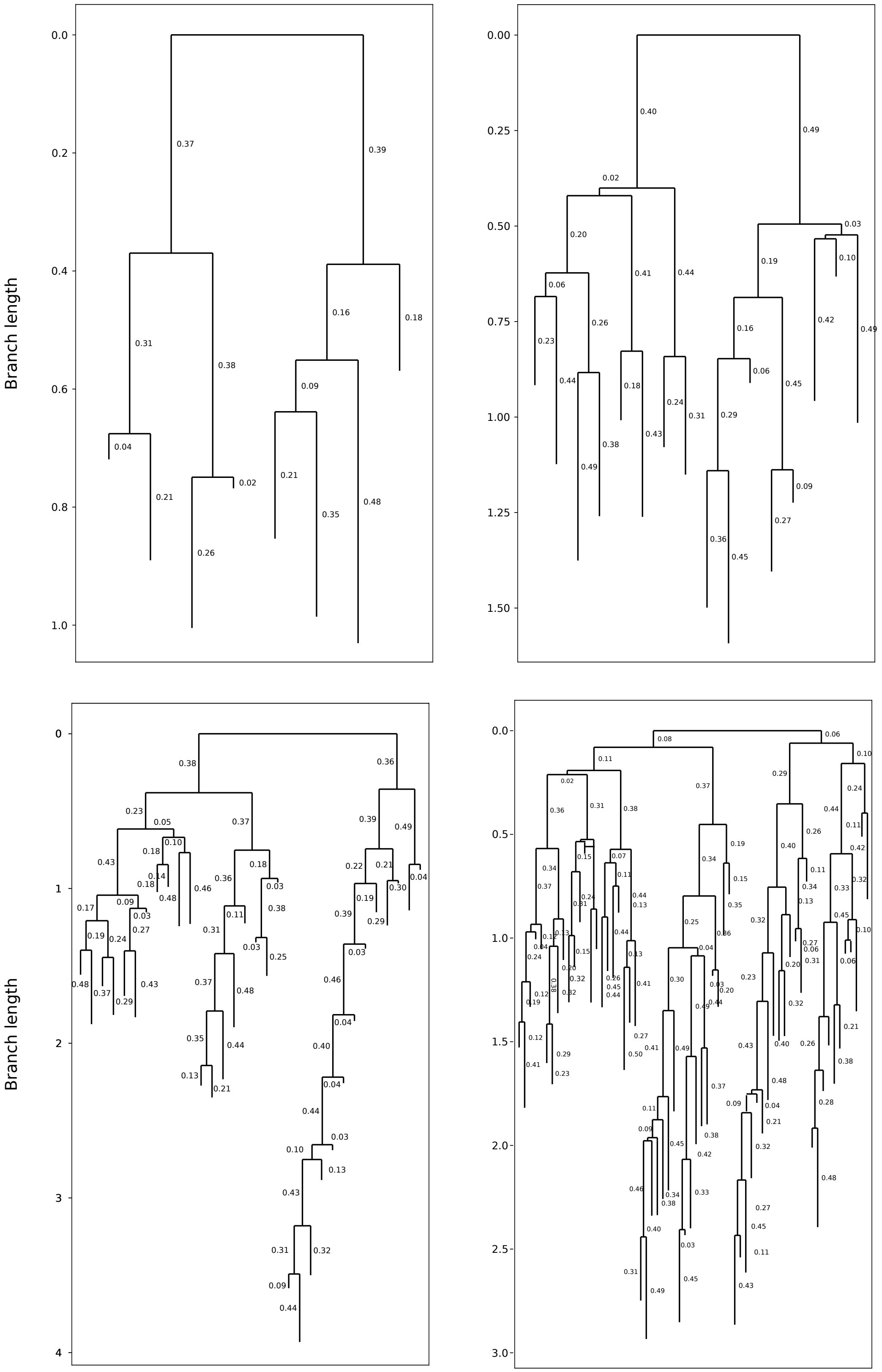


**Supplementary Figure 11:** The four randomly-generated phylogenies (8-, 16-, 32- and 64-taxa; available in Sup. Files) used as guides to simulate internal and terminal node sequences (see *Tree generation and alignment simulation*, *Ancestral state reconstruction*).

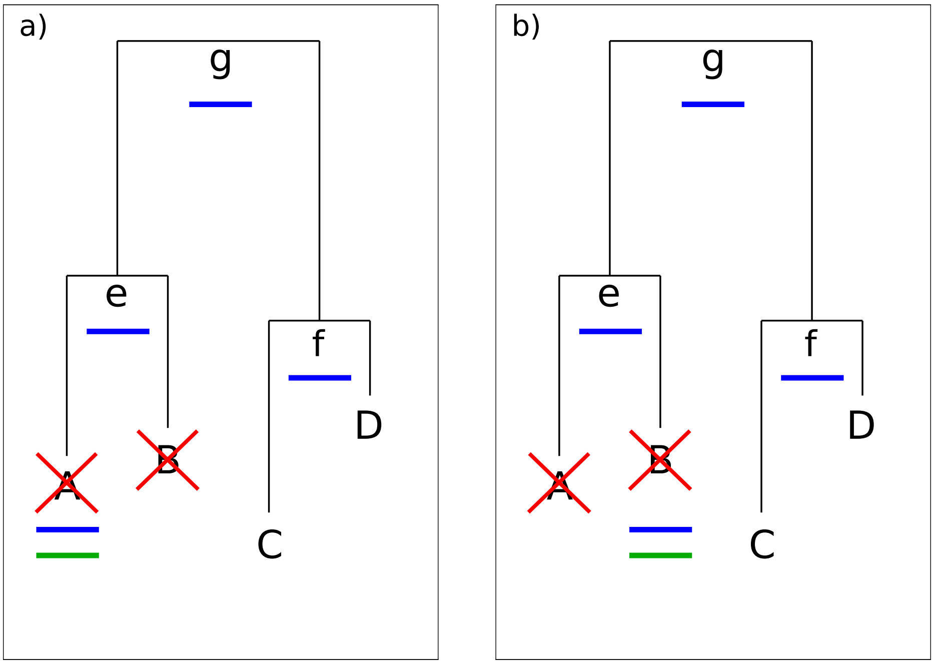


**Supplementary Figure 12:** Illustration of the LLO procedure. LLO is used to evaluate a model's ability to reconstruct terminal nodes' states. **(a)** A pair of sibling terminal nodes (A, B) is selected, their sequences are removed from the alignment (red ×) and then all internal node sequences are reconstructed (e, f, g, in blue) along with A's sequence. This can be compared to its original sequence (green). **(b)** The analogous procedure is then followed to reconstruct B's sequence and compare it to the original.
