# Supplementary figures and images for: Modeling Structural Constraints on Protein Evolution via Side-Chain Conformational States
Source: Mol Biol Evol. 2019 May 22;36(9):2086–103. doi: 10.1093/molbev/msz122 (PMC6736381; doi:10.1093/molbev/msz122)

a)

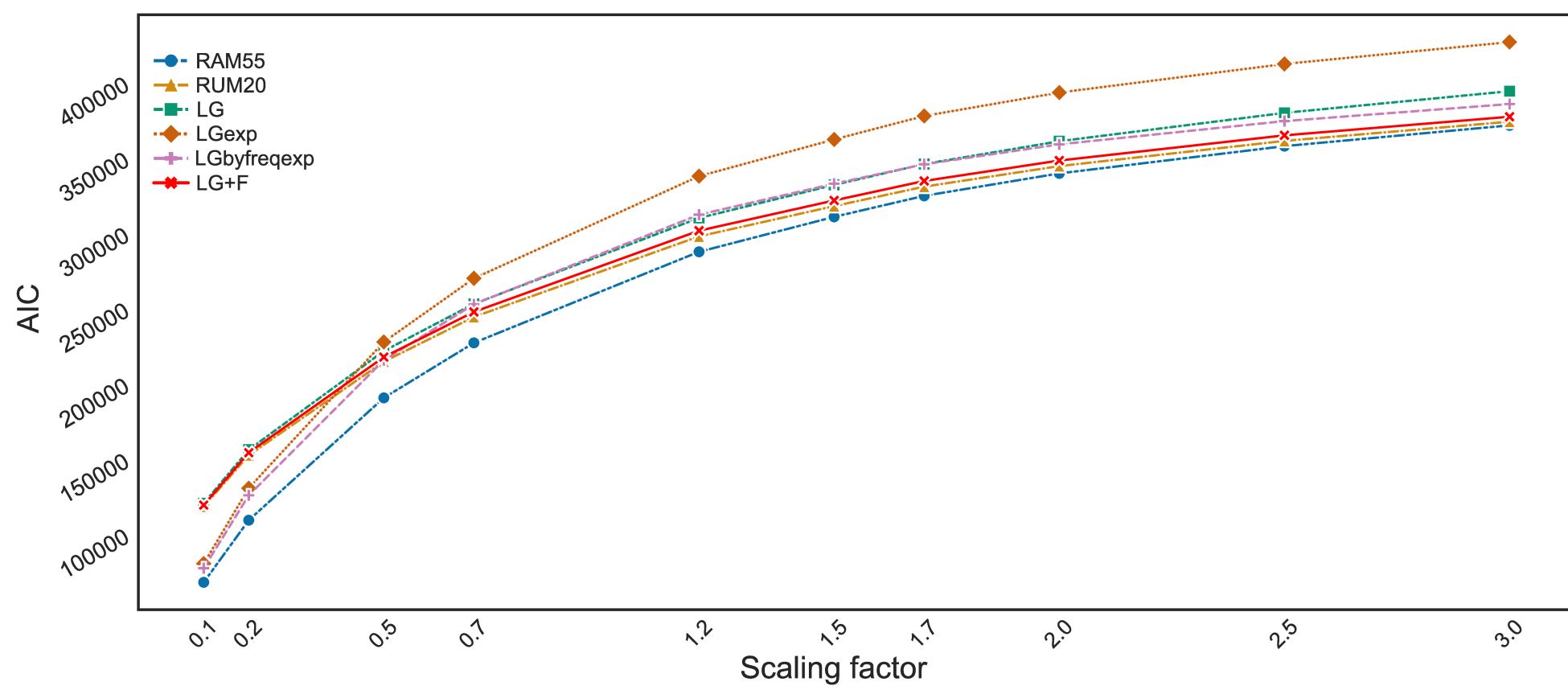

b)

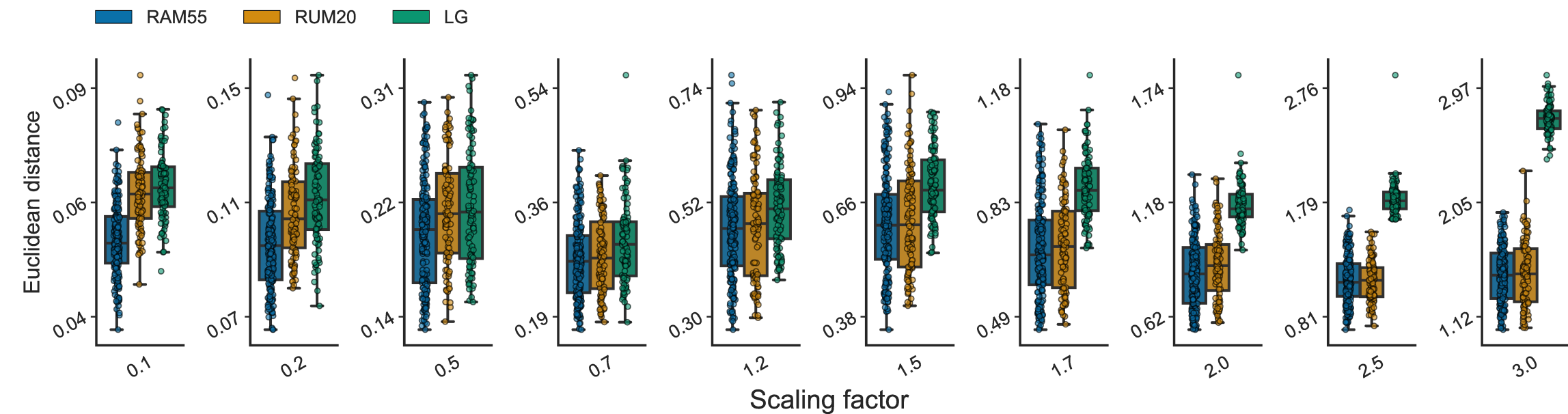

Supplement: msz122_Supplementary_Data [file msz122_supplementary_data.zip › AICvALL_and_euclideanv20x20-LG_64x1000_redo.pdf]

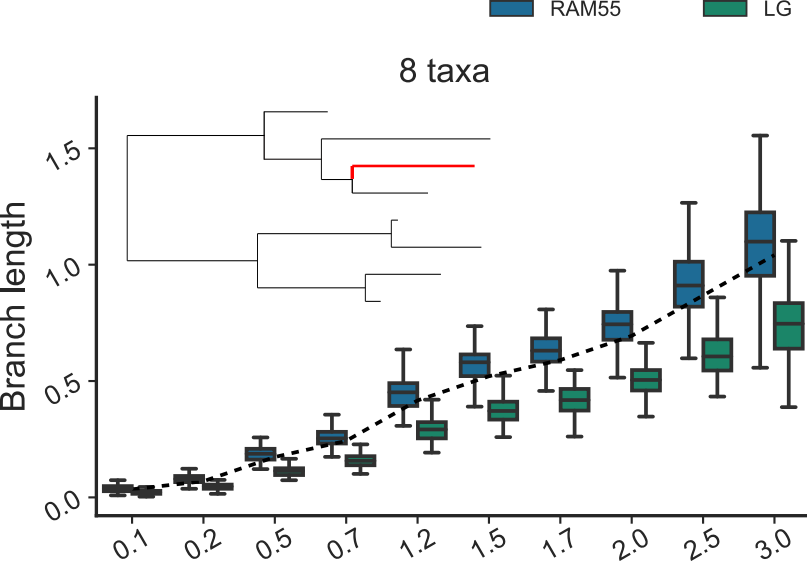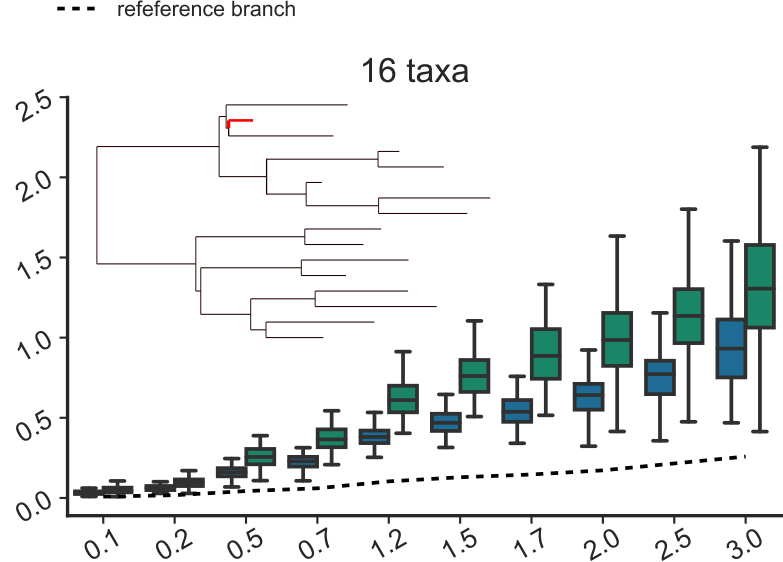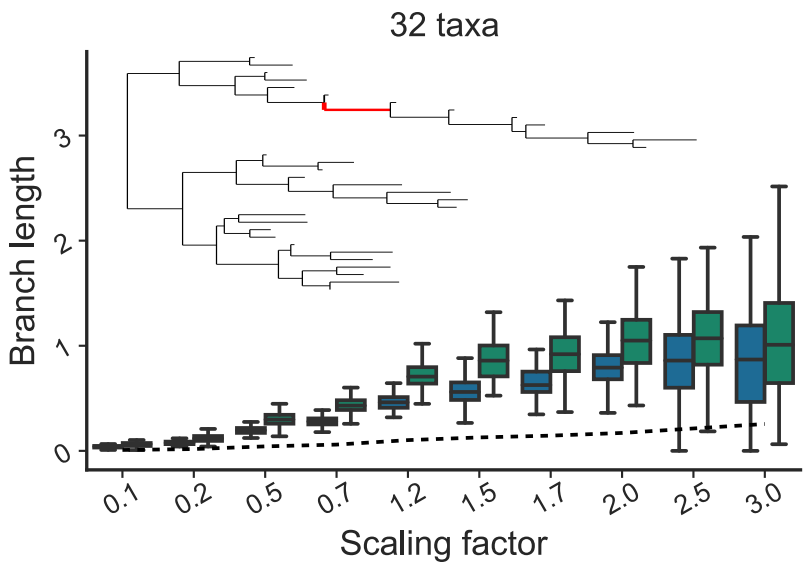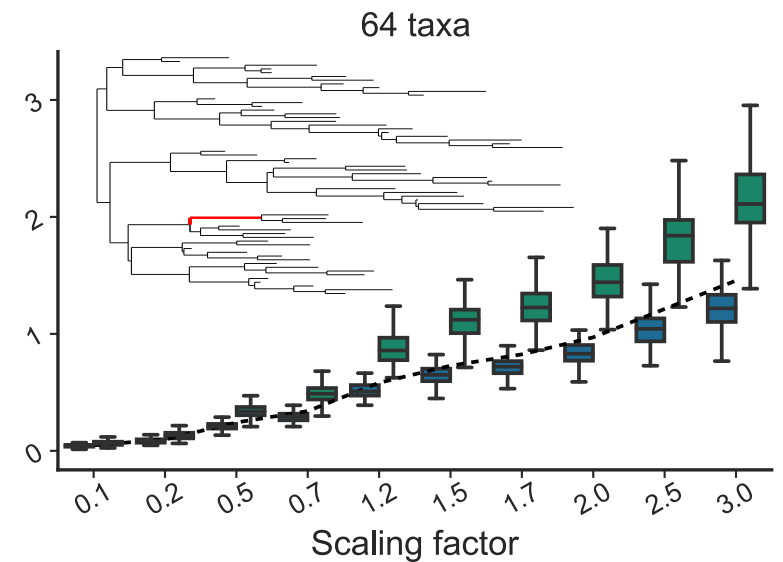

Supplement: msz122_Supplementary_Data [file msz122_supplementary_data.zip › ALLx200x20_simulation_55x55vLG_single-brlen_compare.pdf]

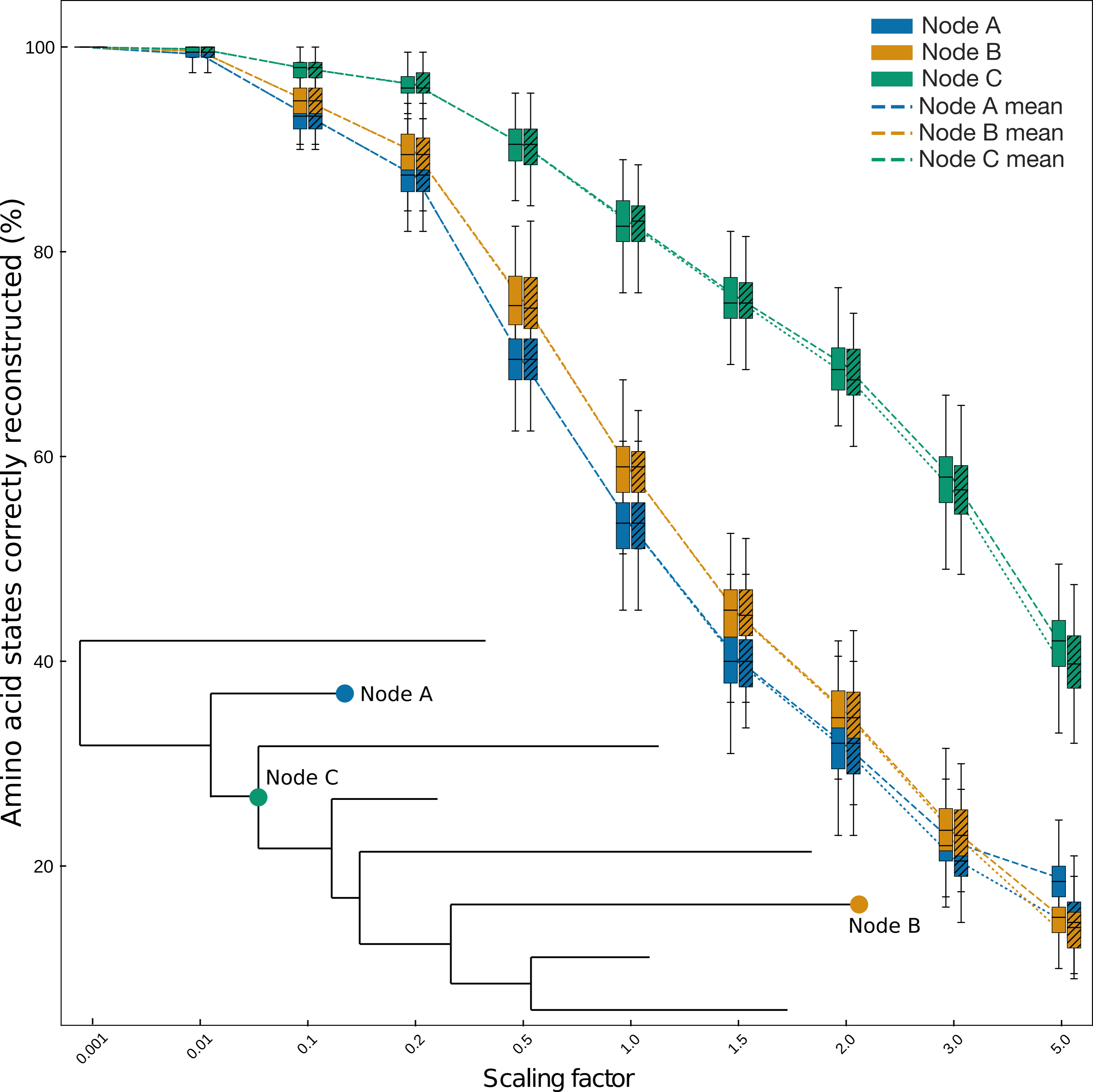

Supplement: msz122_Supplementary_Data [file msz122_supplementary_data.zip › Ancestral_AA_vsLG_simulation.pdf]

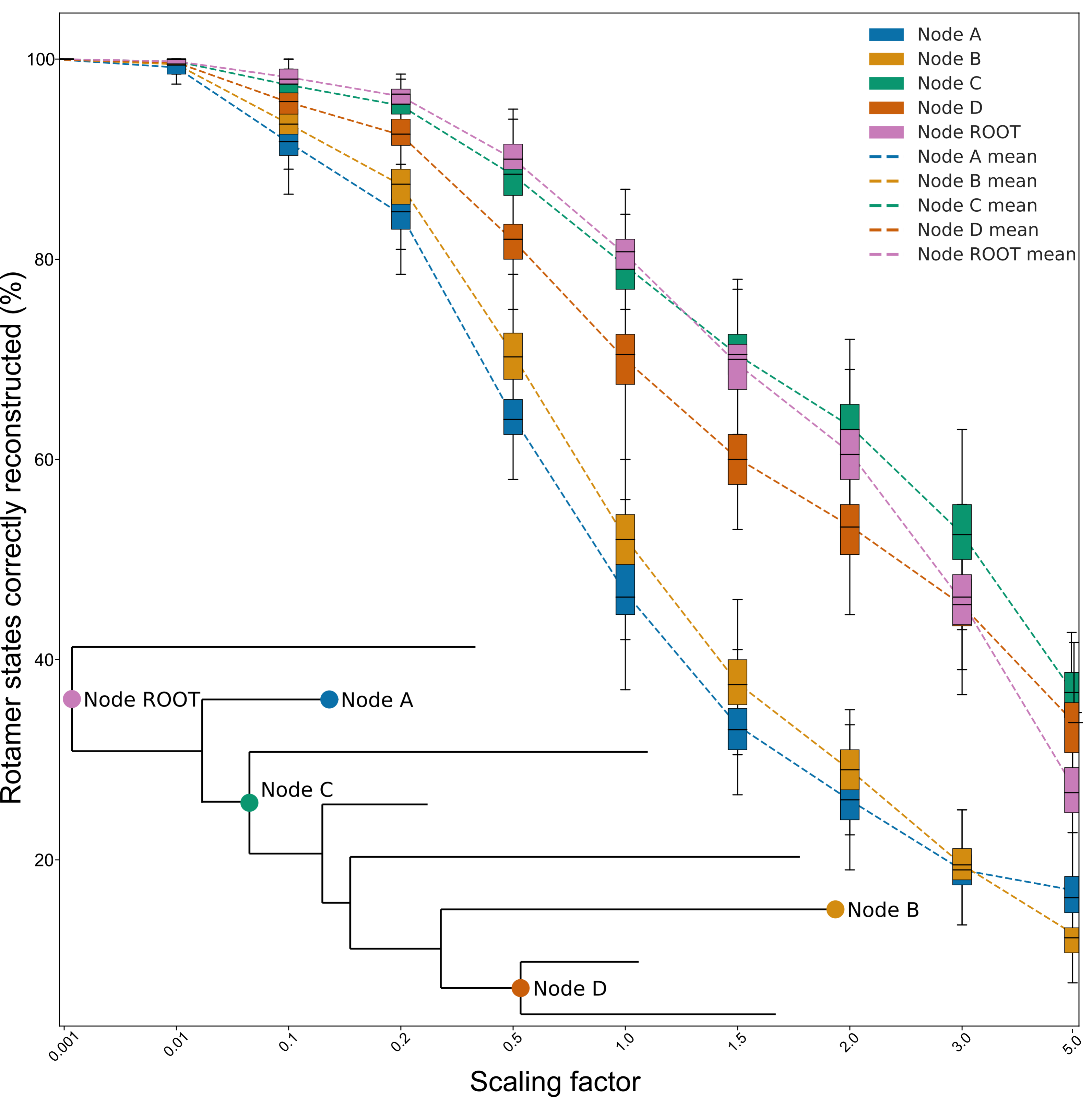

Supplement: msz122_Supplementary_Data [file msz122_supplementary_data.zip › Ancestral_rotamer_55x55_simulation.pdf]

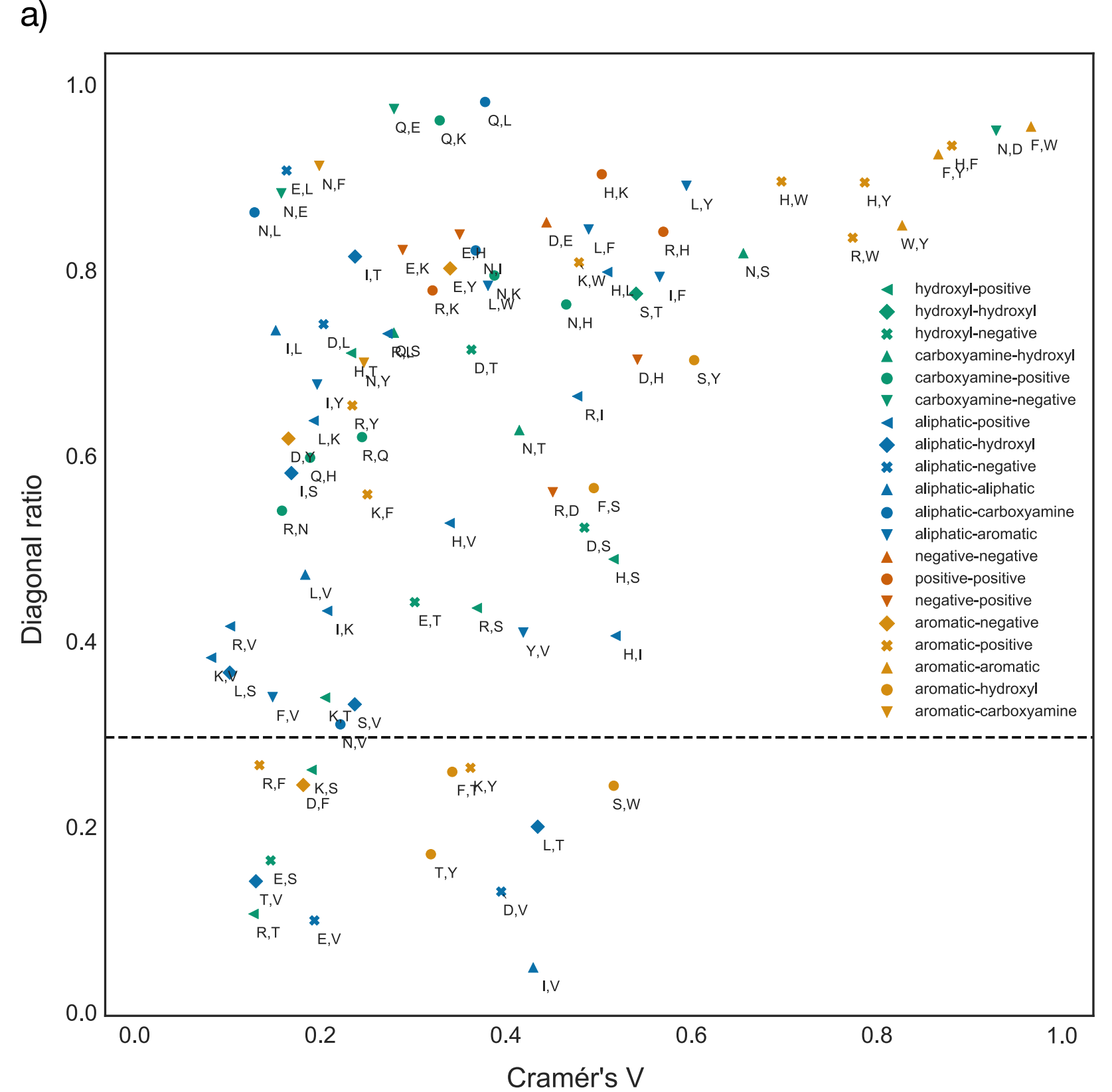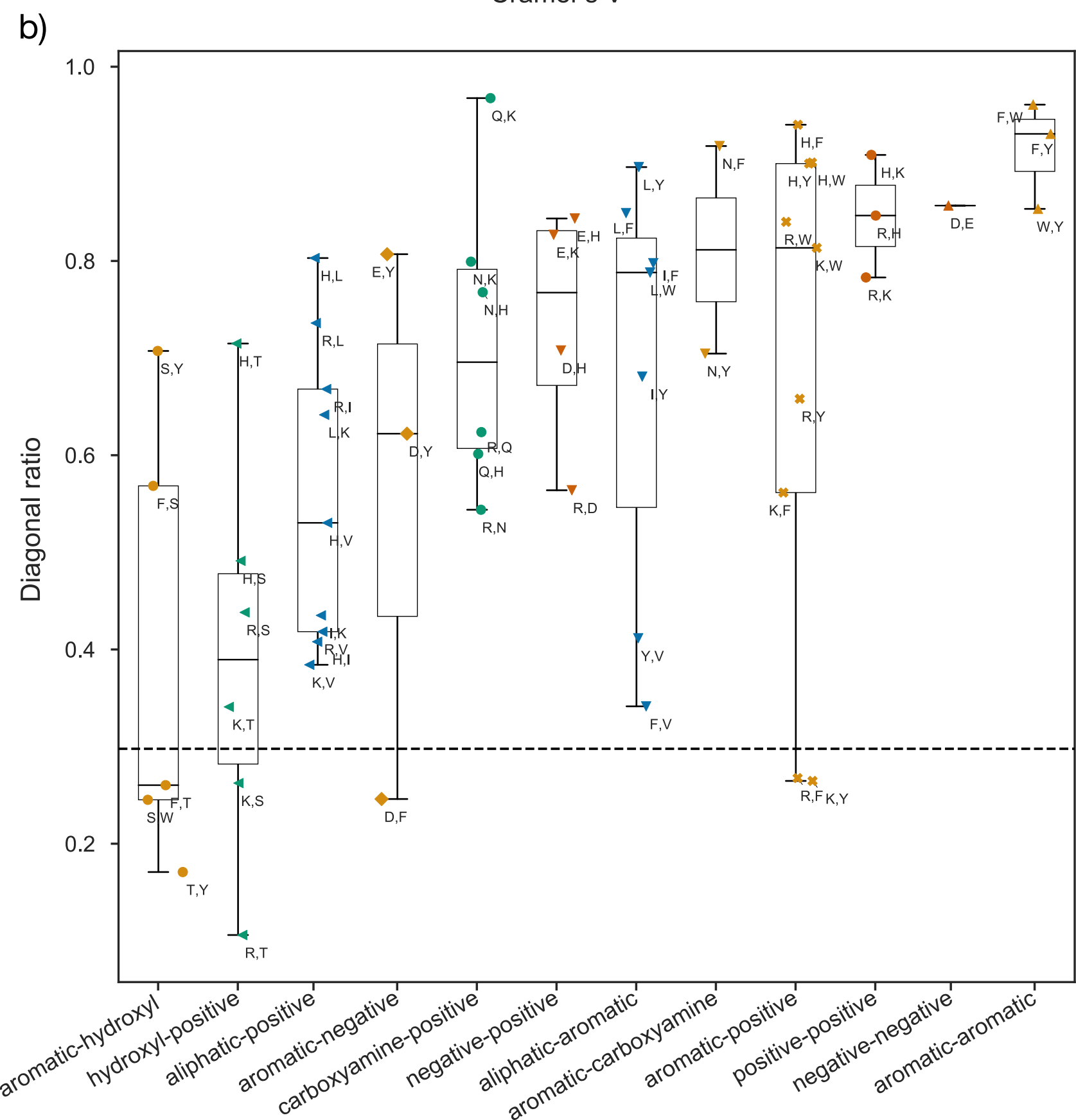

Supplement: msz122_Supplementary_Data [file msz122_supplementary_data.zip › CramersV_diagratio_scatter_and_boxplot.pdf]

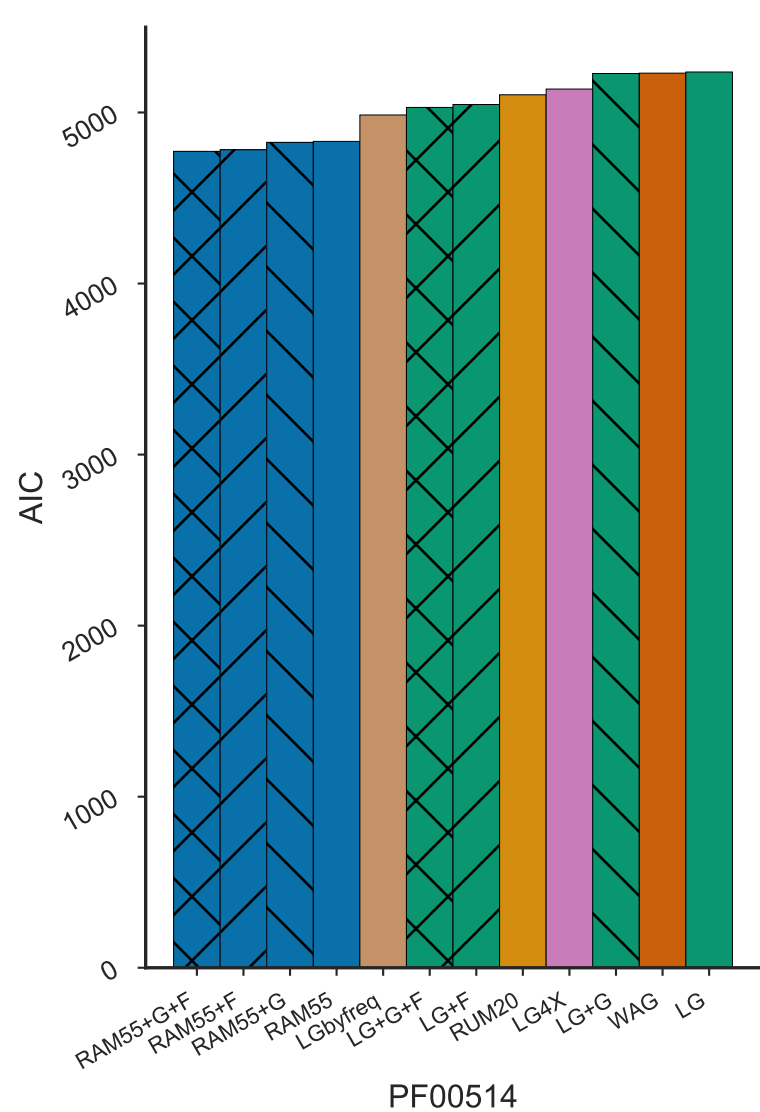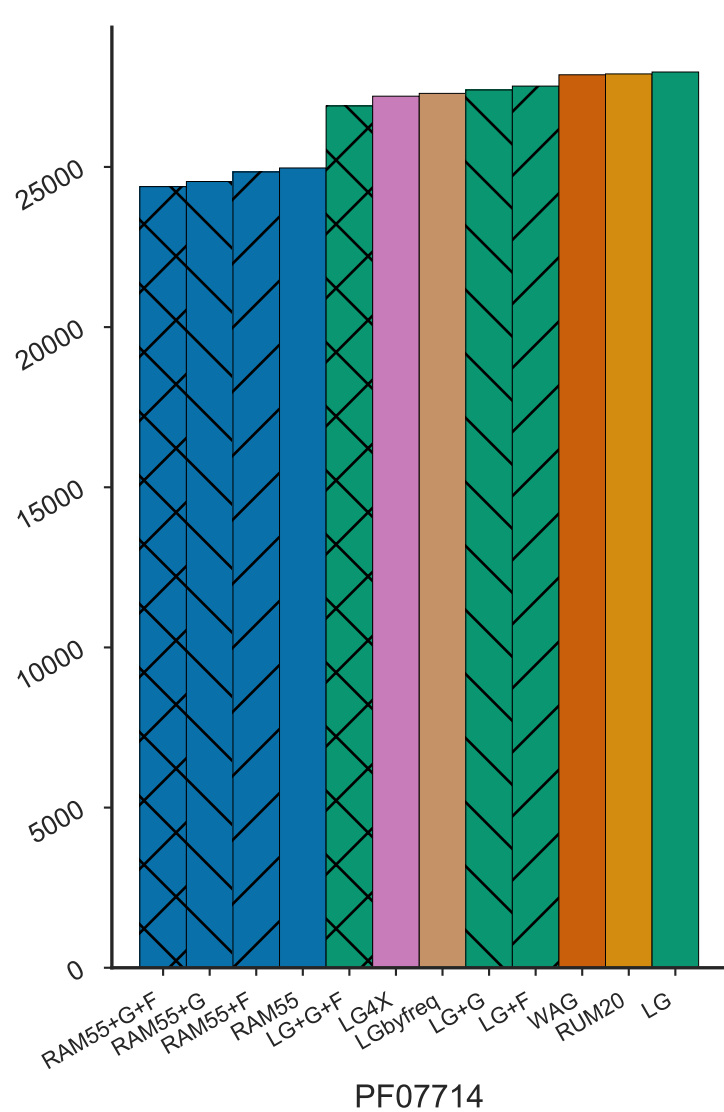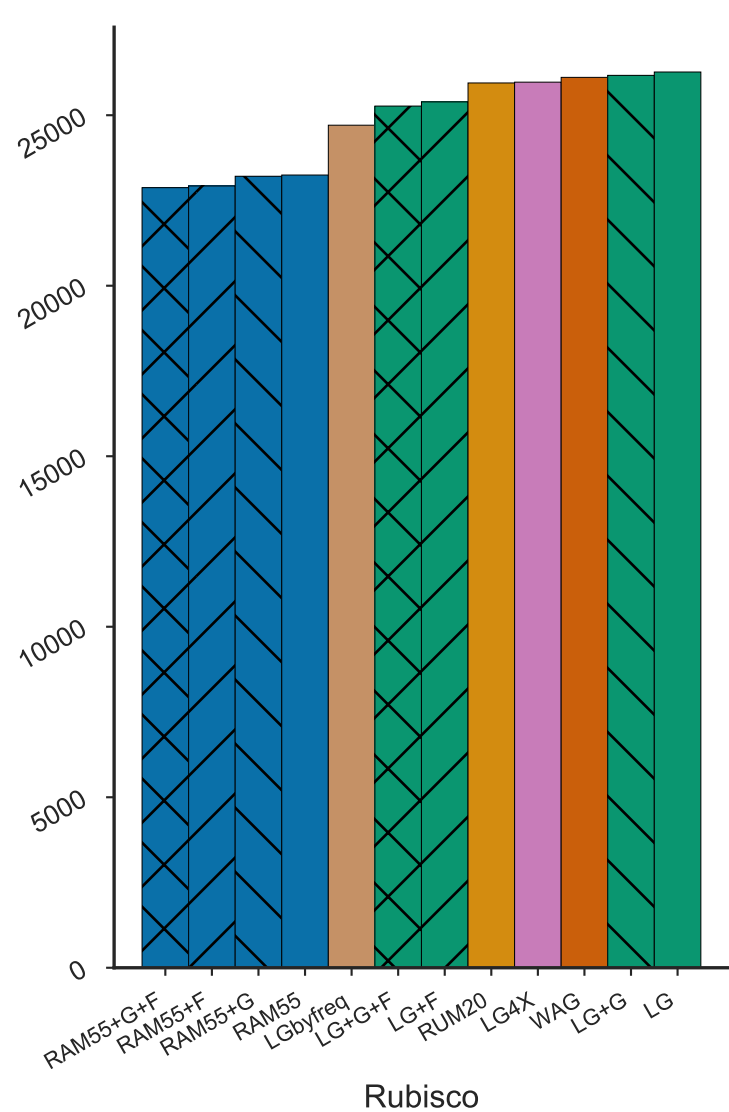

Supplement: msz122_Supplementary_Data [file msz122_supplementary_data.zip › empirical_AIC_histo.pdf]

a)

PHE

TRP

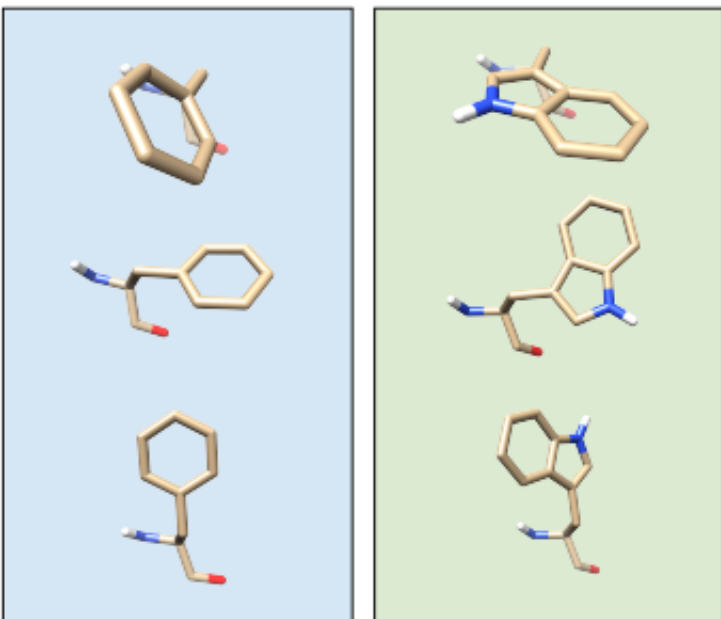

b)

PHE

TRP

1

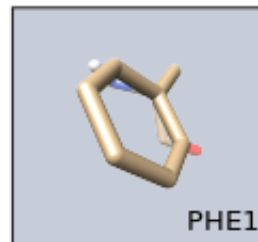

PHE1

TRP1

2

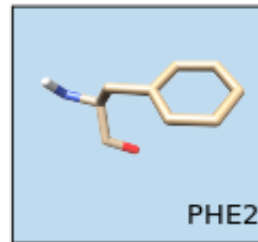

PHE2

TRP2

3

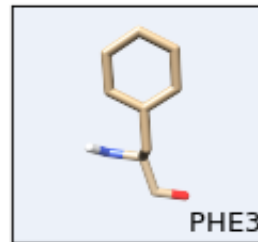

PHE3

TRP3

Supplement: msz122_Supplementary_Data [file msz122_supplementary_data.zip › Expanded_alphabet.pdf]

a)

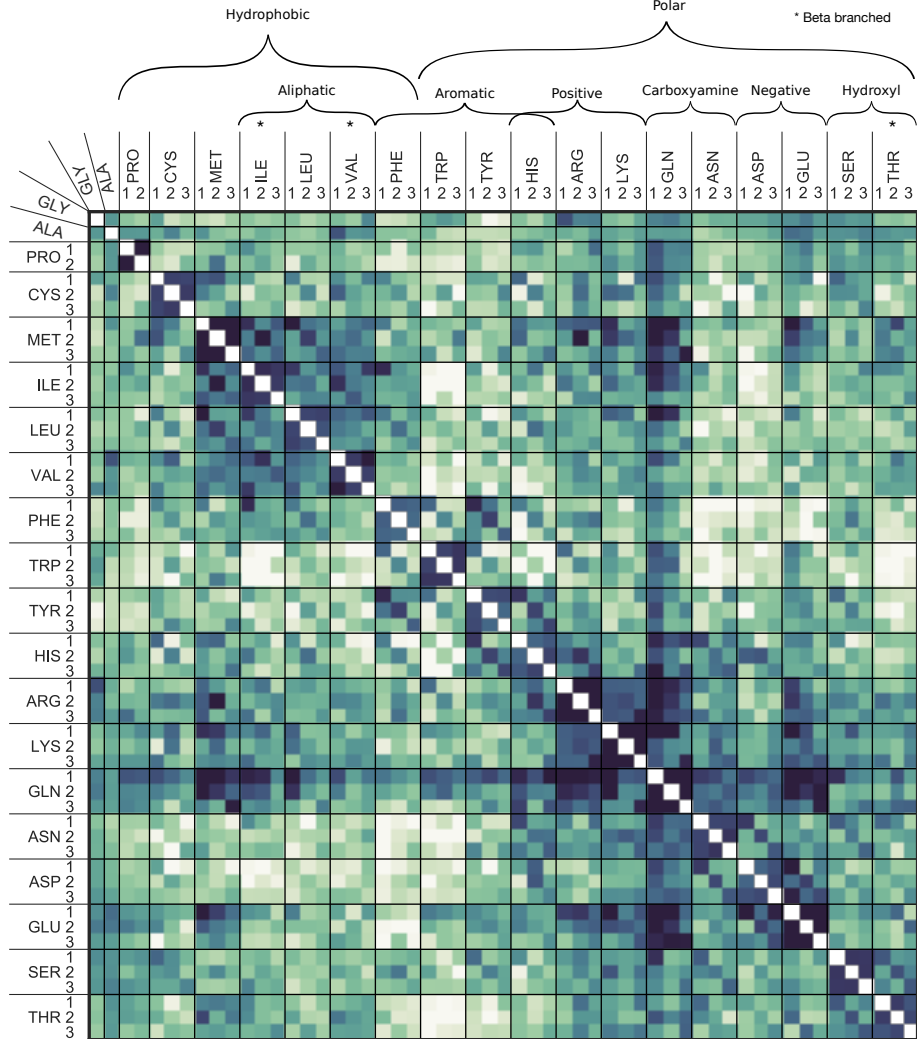

b)

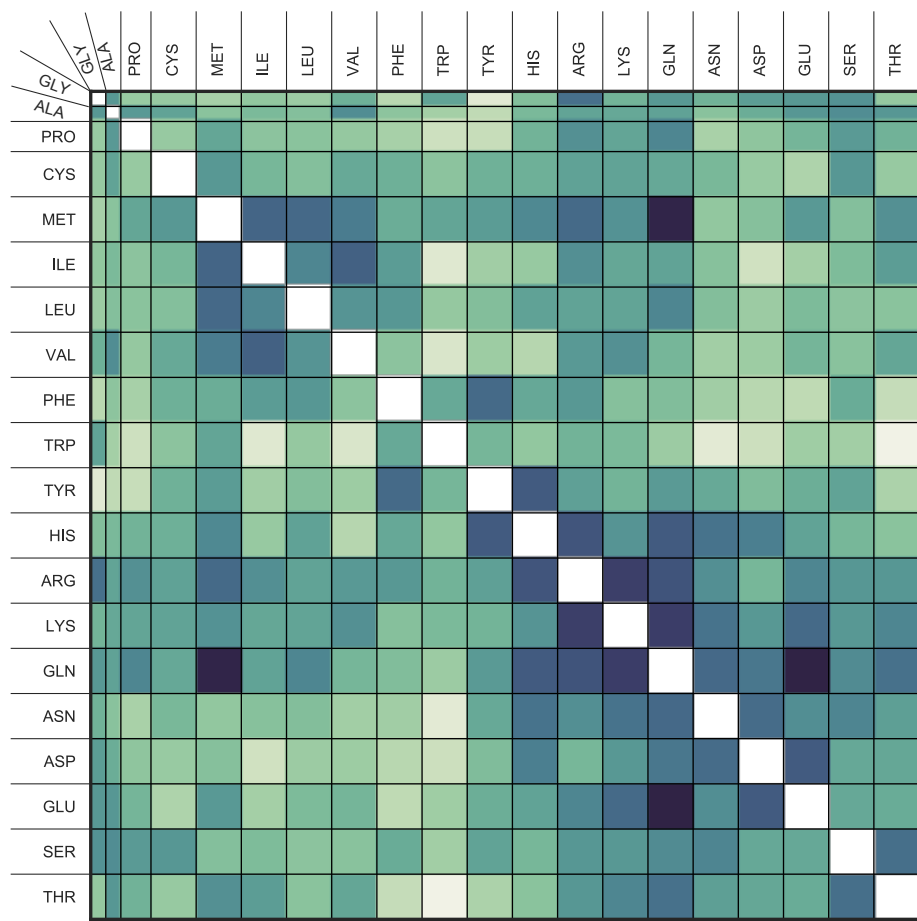

Supplement: msz122_Supplementary_Data [file msz122_supplementary_data.zip › heatmaps_downsized_nofreqs.pdf]

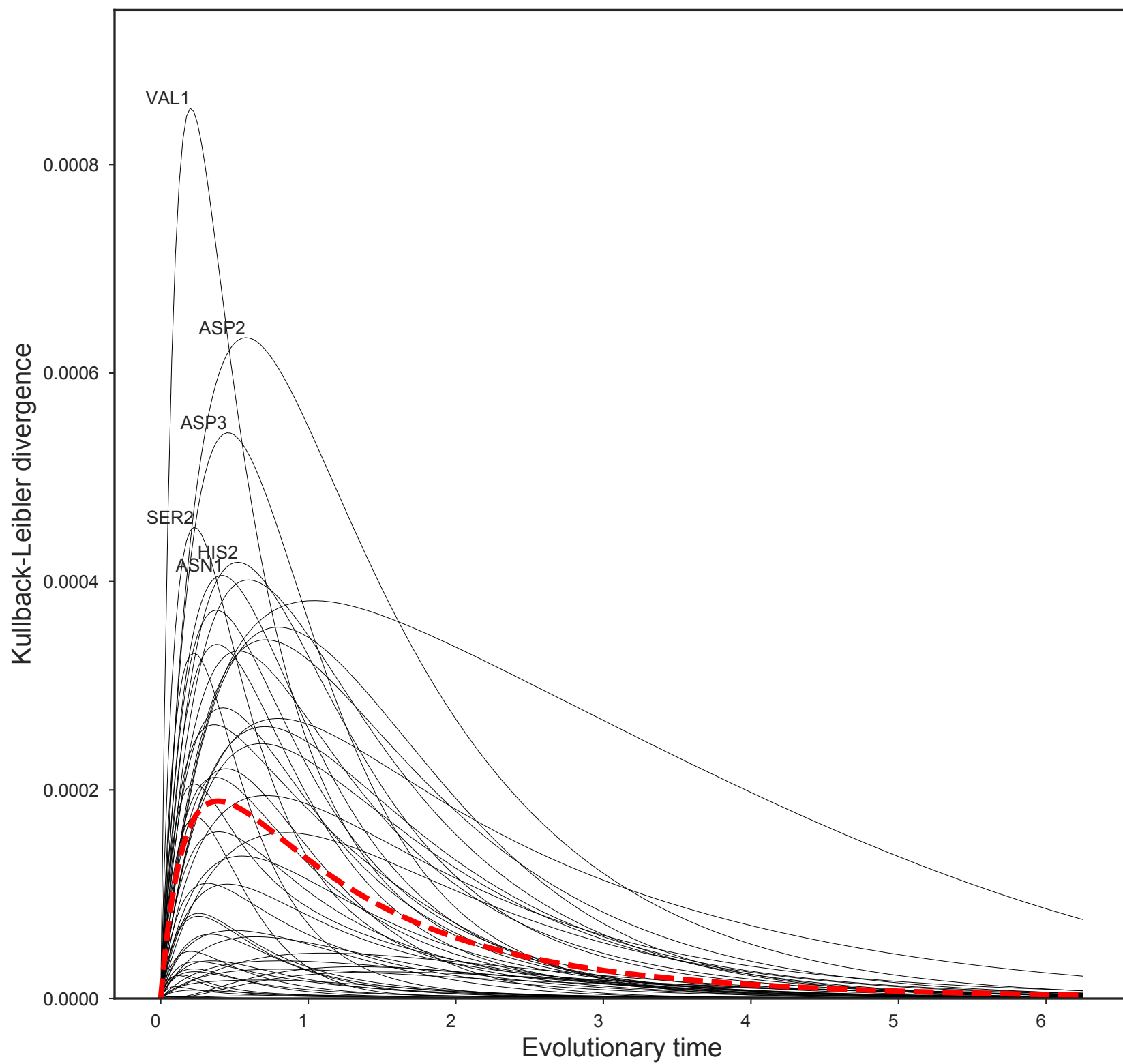

Supplement: msz122_Supplementary_Data [file msz122_supplementary_data.zip › mean-new_KL.pdf]
